# Authorship and ChatGPT: a Conservative View

**DOI:** 10.1007/s13347-024-00715-1

**Published:** 2024-02-26

**Authors:** René van Woudenberg, Chris Ranalli, Daniel Bracker

**Affiliations:** Department of Philosophy, Vrije Universiteit, Amsterdam, Netherlands

**Keywords:** ChatGPT, AI, Authorship, Agency, Assertion, Conservativism, Intention, Normativity, Promising

## Abstract

Is ChatGPT an author? Given its capacity to generate something that reads like human-written text in response to prompts, it might seem natural to ascribe authorship to ChatGPT. However, we argue that ChatGPT is not an author. ChatGPT fails to meet the criteria of authorship because it lacks the ability to perform illocutionary speech acts such as promising or asserting, lacks the fitting mental states like knowledge, belief, or intention, and cannot take responsibility for the texts it produces. Three perspectives are compared: liberalism (which ascribes authorship to ChatGPT), conservatism (which denies ChatGPT's authorship for normative and metaphysical reasons), and moderatism (which treats ChatGPT as if it possesses authorship without committing to the existence of mental states like knowledge, belief, or intention). We conclude that conservatism provides a more nuanced understanding of authorship in AI than liberalism and moderatism, without denying the significant potential, influence, or utility of AI technologies such as ChatGPT.

## Introduction

Consider the following case:

*Assignment*: Alice submitted a final paper that she’s proud of. She thinks that the argument is interesting, insightful, and that its conclusion is plausible. The teacher agrees: she gives Alice’s paper an A+ with the comments: “Amazing. I don’t know how to improve on this paper—I feel like I learned something interesting and important”.

The case features an evaluation. The teacher evaluates Alice’s paper as excellent. She could see its excellent features and even learned from the paper. There’s nothing astonishing or controversial about this kind of evaluation. *Of course* some final papers are crafted so well that it moves the teacher to judge that the work is excellent. *Of course* some papers are so careful, well argued, and insightful that we think it’s author must be experienced and talented. We all know this.

Now here’s the twist. Imagine that Alice is not responsible for the text; her creative writing played no role. Instead, ChatGPT produced the paper. Indeed, some ChatGPT produced works have gotten past expert blind-review (Else, 2023), have earned college students’ first-class grades (Wild, 2023), or even won artistic awards (Grierson, 2023). But these decisions were all made without the evaluators knowing that ChatGPT was the author.^1^

*Or was ChatGPT the author*? That’s our main question. Authorship and related concepts like ‘composer’, ‘poet’, ‘novelist’, and so forth all seem to encode information about creative, deliberative agency. What’s puzzling is that when we make epistemic, ethical, or aesthetic evaluations about texts, we often seem to be making evaluations about their authors. For Alice to be the author of her paper, didn’t she have to know or at least believe its content? But how could ChatGPT have the requisite knowledge or belief if it doesn’t even have mental states?

The *Assignment* case helps to bring out certain other discursive expectations we have of authors as well. The teacher evaluated the essay epistemically, aesthetically, but also formally, namely, how well it met the criteria set out for the assignment (e.g., clarity, argumentation, creativity, structure). But then, supposing ChatGPT is the author, it must have *intended* to meet the assignment’s criteria—or even exceed it. When we evaluate ‘Alice’s paper’ for excellence, our praise is directed at what *she authored*; her craftwork, creativity, and ability to meet the assignment’s standards. What’s more, we may turn to her for further explanation; we can ask her: “what did you mean here?” or “why did you write that?” and expect clarification, justification, and, if there was a mean statement in the text, regret. But with ChatGPT as the source, these expectations break down. What would it mean to praise ChatGPT for its effort, or expect remorse or compensation for its offense? How could we even demand a justification from ChatGPT?^2^ How could we expect compensation from *it*?

The *Assignment* case reveals a puzzle, then. On the one hand, the evaluations of Alice, based on her submitted assignment, seem natural when there’s what we later call ‘normal authorship’, i.e. when she wrote the text herself. On the other hand, when we swap the author with ChatGPT, we can no longer make sense of such evaluative practices without error, confusion, or serious revision. For this is also puzzling. If there’s no difference in the texts produced but only their source, then perhaps our evaluations should remain; wouldn’t it be too rigid to take back our evaluations upon learning that the texts had an artificial (albeit arguably intelligent) source?

This puzzle might be thought to instance a more general problem. The relevant kinds of evaluations each seem to reflect properties that only *agents*, in their capacity as authors could possess. Authors have intentions, beliefs, reasons, expectations, and pieces of knowledge, which facilitate the creation of their work. Moreover, authors can be morally or epistemically responsible for their work; they can be praiseworthy or blameworthy for failing to attend to the relevant evidence, or praised for their acute perceptiveness, for example. In general, they are answerable to others for their authorship. But with ChatGPT as the source of the relevant texts, there’s just no clear agent (or something agential) to bear the benefit (or the burden) of our evaluations. This creates an *attributability gap*, echoing similar worries about responsibility gaps for AI in general (see Santoni de Sio & Mecacci, 2021).^3^

How should we respond? Some might argue that the relevant kinds of evaluations of texts all require normal authorship and so a certain kind of agency, but that ChatGPT lacks agency, rendering our initial (pre-reveal) verdict about Alice strictly mistaken. Call this the *conservative view*. Roughly put, the argument for the conservative view is that our evaluations are fundamentally about properties traceable to authorship, and that claims to authorship stick only when certain mental states and normative capacities are present. That’s what’s missing in the case of ChatGPT textual production, however, and so it doesn’t really ‘author’ anything. *Mutatis Mutandis* for any other LLM currently lacking strong general artificial intelligence.

However, others might contend that, in light of the emergence of sophisticated LLMs, such as ChatGPT-4 or models like Microsoft and Meta’s Llama 2 (utilized in platforms like Azure and Windows, or PaLM which powers Google’s chatbot Bard), that we must revise our views of authorship. Although initially we may have thought that only human beings could author novels, essays, poetry, grants, or research articles, LLMs force us to question this thought and so the attendant presuppositions which underly it. Perhaps ChatGPT *intended* to satisfy the requirements for an A-grade. Maybe ChatGPT *knew* or had *justification to believe* that, say, its research proposal would be competitive. And so on. Call this the *liberal view*.^4^ The argument for the liberal view, in a nutshell, is that it can parsimoniously account for ordinary language surrounding AI.^5^ Consider the seeming propriety of statement *(A)* “You didn’t author that essay, ChatGPT did”. The truth-conditions of this sentence seem to be that there is something which authored an essay, that it is ChatGPT, and that if it is ChatGPT, then it wasn’t you. If the conservative view is correct, then *(A)* is false, because ChatGPT simply lacks the properties and capacities necessary for authorship. So, assuming the propriety of statements like *(A)*, the conservative view faces the burden of explaining or dispelling the propriety of *(A)*. On the liberal view, by contrast, *(A)*’s propriety needs no explanation. (We will present the conservative’s account of *(A)*’s propriety in §5.)

Between the conservative and the liberal there is a *moderate view* according to which conservatives are wrong to think that ChatGPT and other new deep learning AI aren’t authors, but that the liberals are wrong to think that ChatGPT and its kin should be attributed mental state-types of the sort routinely ascribed to humans, like knowledge, belief, intention, and so forth. In turn, they’ll deny that ChatGPT bears knowledge-, belief-, or intention-entailing relations, like promising, assertion, or assurance as well.^6^

The main argument for the moderate view is that it avoids the psychological sectarianism of the conservative view and the ontological excesses of the liberal view. In due course, we’ll explore in some detail how moderates might defend this claim, but as a rough preview, moderates can say that we should adopt the ‘intentional stance’ towards ChatGPT, and thereby treat ChatGPT *as if* it had beliefs, knowledge, and intentions; or else moderates could give a *functional* account of what performs authorship-roles, so that authorship only requires performing certain functions, like generating a text that is coherent, contextually appropriate, and even novel, or otherwise enrich our conception of authorship without taking on the burdens of either conservative or liberal ontology (see, e.g., Floridi, 2023).

We argue in favor of the conservative view. Conservatives must explain the apparent propriety of statements like *(A)* without demanding retraction or serious changes in the concept of authorship. Moreover, conservatives need to explain why liberals and the moderates aren’t right and they should do so without succumbing to AI-chauvinism or a romantic vision of the human person. That’s what we try do in this paper. In turn, the conservative view doesn’t demand *skepticism about AI*, specifically that AI cannot in principle be an author, an agent, a knower, and so forth.

Our argument proceeds as follows. First, we outline some preliminaries about ChatGPT and authorship (§2). Second, we detail what we call ‘normal writing’ which we argue, is undetachable from agency and its normative features (§3). This leads us to the epistemic and practical functions of authorship-ascriptions, which we argue are agency-entailing, with a certain mental state ontology (e.g., belief, desire, intention, knowledge) (§4). We then zoom in on relevant cases, like promising and assertion, which we argue ChatGPT is incapable of performing (§5), before considering the shortcomings of the liberal and moderate views (§6). We conclude with some considerations about deep-learning AI and open-questions about epistemic attributability gaps.

## Preliminaries

What is ChatGPT? It is a deep learning natural language AI system that generates something that reads like a human-written text. Let us say neutrally that it ‘generates texts’.^7^ And it does so given certain instructions and prompts that users prompt the system with. For example, the prompt “Tell me about Martin Luther King” may generate a textual output with key facts about Martin Luther King, or major events involving Martin Luther King.

Two things about ChatGPT are especially important to keep in mind. First, as Stephen Wolfram explains, “what ChatGPT is always fundamentally trying to do is to produce a ‘reasonable continuation’ of whatever text it’s got so far, where by ‘reasonable’ we mean ‘what one might expect someone to write after seeing what people have written on billions of webpages, etc.”^8^ In other words, what ChatGPT is essentially doing is ‘asking’, over and over again “given the text so far, what should the next word be?”, and each time adding a word. The ‘answer’ is based on the calculated probability that the next word in the sequence *ABC* (where *A*, *B* and *C* are words) is *D*.

Second, even if ChatGPT produces a sentence that is true, it is not necessarily ‘aiming’ at truth. By this we mean that even if the output sentences are true, or the output text as a whole is accurate, this is an unintended by-product of ChatGPT’s computations. Truth is not a design principle of ChatGPT, as it was of the SHRDLU AI system. Of course, whether this means that relying on ChatGPT-generated texts for belief renders the corresponding belief unjustified, irrational, or incapable of producing knowledge is a further question.

## The Conservative View

### Normal Writing

What is the connection between writing and authorship?^9^ We begin by discussing the nature and normativity of what we call ‘normal writing’. Normal writing has the mark of intentionality. It is an *intentional* action in five different senses.^10^ Writing is, first, an intentional action, i.e. it is not due to chance, it isn’t a fluke or a coincidence that one writes when one writes. Persons who write normally have the intention, even the conscious intention, *to* write. When one is executing an *intended* action, one normally *knows* what one is doing.

But things aren’t always normal. We use the qualifier ‘normal’ to accommodate two phenomena: (a) Deeply confused persons who write without having the intention *to* write. (b) There is also writing in trance, or under hypnosis. These are clear exceptions to what normally is the case when one is writing.

Second, writing is normally also intentional in that authors intend to write about *something in particular*. Normally people who write know *what* they are writing about—about the latest Nobel Prizes, for instance, or about Darwin’s voyage on the Beagle.

The ‘normality’ qualifier accommodates (c) cases of deeply confused person who may know that they are writing, even if they have no idea what they are writing about. (d) Cases of people involved in forms of *écriture automatique*, i.e. people who know they are writing, but do so without conscious policing their thoughts, without letting what they write be influenced by morality or by what is politically correct.^11^

A third way in which writing is intentional is that it is normally directed at others, it *targets other persons*. When one writes, one normally wants to communicate something to others: what one writes is normally *addressed*. The address can be a very specific person, or a well-defined set of persons, but also less well-defined groups, and even only merely *hoped-for* readers.

The ‘normally’ qualifier also accommodates (e) cases of exercising one’s writing hand or typing skills and so writing *without addressing anybody* and (f) cases of making notes for oneself only, and so not targeting other persons, but oneself at a later time. Normally, however, when one is writing there are *others* who are targeted.

Writing is furthermore intentional in that it is normally *about* things. What we write is normally about something, like, politics, poetry, or psychiatric disorders. The products of normal writing, texts, have what Franz Brentano called intentionality: they have *about-ness*.

The ‘normally’ qualifier also accommodates (g) cases of confused writing that are not about anything due to a psychological condition like Alzheimer’s disease and (h) cases of delightful nonsense poetry that lack propositional content.

A final way in which writing is normally intentional is that *through* writing, persons normally attain, and intend to attain, aims that are external to the writing. For example, writers intend to inform their readers, motivate them, interrogate them, warn them, entertain them, etc. Normally writers have illocutionary and perlocutionary intentions.

The qualifier accommodates cases of writing without intending any effect in possible readers; the writing *is* an end in itself, performed “just for the fun of it”. Putting these points together we define ‘normal writing’ as follows:

A piece of writing *W* is ‘normal’ when: (i) there is an author who intentionally writes *W*, i.e. knows *that* they are writing; (ii) the author intends to write (about) something in particular, i.e. knows *what* they are writing; (iii) the author addresses or targets a readership with *W*; (iv) *W* has intentionality; (v) the author intends to secure some effect in their readership through *W*.^12^

But something should be added. St. Bonaventure called an *auctor* someone who writes “his own words”. He distinguished *auctors*, from *scriptors* (i.e. someone who copies texts), from *compilers* (i.e. someone who puts passages together from texts that are not his own), and from *exegetes* (i.e. someone who adds his own words to the words of others.) It seems clear that neither scriptors nor compilers, although they write, are authors, since they don’t engage in normal writing. For normal writing requires, to use Bonaventure’s phrase, that one writes one’s own words. We could put this as follows: someone’s writing is normal writing when the writer is the content owner of the writing. This must be added as element (vi) to the definition of normal writing. For the purposes of this paper, we assume that (i), (ii), (iv), and (vi) are essential to normal writing, whereas (iii) and (v), strictly speaking, although they are usually present in normal writing, are not. Given this explication, we now define an author as someone who engages in, or has engaged in, normal writing.

### Forms of Authorship

There are various ways in which people can engage in normal writing. One way is to write out the text oneself, through handwriting, keyboard input, or voice-to-text, as when one dictates a short text to the note app in their phone.

Secondly, there is what Nicholas Wolterstorff has called authorial ‘superintendence’,^13^ a phenomenon that comes in degrees: there is dictation to a secretary, which may be a line-by-line voice-to-text production, or close to it, with superfluous and pausing words like ‘um’ or ‘uh’ omitted; next a mere indication of substance to a secretary (e.g., “Tell them that there is a meeting tomorrow morning when we get in” becomes the text “Dear development team, there is a meeting tomorrow are 9:00 a.m.”); next there is writing by a secretary without such indication as when the secretary ‘knows the mind’ of the executive, and who accordingly qualifies as the author.

Third, there is also authorship by what Wolterstorff calls ‘authorization’: appropriating someone else’s text, letting it serve as a medium for one’s own communication.^14^ Examples like the following make this plausible. If a snail on the beach leaves a trace behind that reads like the sentence “I love you”, then if you see that snail produce that trail, you wouldn’t believe that you are reading a declaration of love. But if you next see that your partner adds the words to the sand “Really! —Elizabeth”, then you normally would believe that. Or suppose you feel down and full of self-doubt and while shopping, you see a reck of gift cards that carry texts like “You can do it!” and “You’re awesome!” then normally wouldn’t feel encouraged, or reassured. Such effects, normally, can only be hoped for when such cards are signed by someone you know, and who appropriates the text.^15^

### Functions of the Ascription of Authorship

What does the concept of ‘author(ship)’ allow us to do? Why do we talk about authorship, and what are the functions of ascriptions of authorship?

The core function of the concept of ‘author’ is that it allows us to assign responsibility to someone for the existence and content of the text. And this, in turn, allows users of the concept of ‘author’ to do at least five things.^16^ First, as noted above, on the basis of what the recipients read, it allows them to ground an evaluative assessment of the author. It allows them to say that the author was correct or mistaken, convincing or not, etc. Ascription of authorship thus play what Fischer and Ravizza call the ‘ledger function’, which functions to track and maintain a report on authors. This helps us to make more general normative evaluations of authors, as when a critic reviews ‘the work’ of a novelist rather than a single text.^17^

Second, as we have flagged in our discussion of *Assignment*, the concept of ‘author’ allows us to determine who must be able to explain and justify what is written. The notion of an author thus plays what has been called an ‘answerability function’.^18^—This enables us to identify who ought to provide answers to questions readers might have about what the text is supposed to mean, or about why what it says is supposed to be justified.

Although the answerability function and the ledger function may sometimes overlap, they are not the same. The role of the answerability function is not to evaluate authors as such, but to help us keep track of who should answer for a text, who must be held responsible for explaining and justifying its content.

Third, the concept of ‘author’ enables us to determine what treatment the person who wrote the text is due, what, given what they wrote, would be just or fair responses. The concept thus plays a ‘desert function’. Given the content of the text, an author may deserve praise, or deserve a grant or even the Pulitzer Prize, but they may also deserve derision, polite silence, or even punishment.

Fourth, the concept of ‘author’ enables us to determine how serious the message/content of the text should be taken. If we can reliably identify* S* as the author of a text, this enables us to evaluate how seriously we should take the text. If *S* is the President of the United States and their text declares war on Iraq, then we should take this much more seriously than if *S* is your little niece. Certain professional roles, then, can further enrich the role of authorship in a given context, as authors may have institutional positions that can lend authority to their writing, as Presidential declarations, professional medical reports, or legal judgments do. This we call the ‘gravity discerning function’ of the concept of author.

Fifth and finally, the concept enables us to determine who should be compensated, or who should compensate others, because of the text. Authorship contracts specify receivers of royalties. And in case the text is offensive to third parties, it enables us to specify who will have to compensate for damages, be it an apology, a retraction, or financial compensation. This ‘compensation function’ is especially prominent in legal contexts, when the texts instance libel, plagiarism, false rumors, or outright lies.

## ChatGPT and Authorship

In light of what we have said about normal writing and authorship, we now turn to ChatGPT and address a number of related questions.

### Do ChatGPT Generated Texts Have Authors?

The basic conservative argument is this:

P1. If ChatGPT authors its texts, then it has the normative, agency-entailing features relevant for author functions.

P2. But ChatGPT lacks the normative, agency-entailing features relevant for author functions.

Therefore,

C. It’s not the case that ChatGPT authors its texts.

It follows that ChatGPT is not an author since there are no other texts for which ChatGPT stands a chance at authoring beside the texts it produces.

Is the argument sound? The first premise is an instance of a plausible general principle about authorship, namely that if *S* is an author, then *S* has the normative, agency-entailing features relevant for normal writing and other authorship functions, as we’ve seen. At root, an author is someone who engages in normal writing (§3.1–3.2), and normal writing embeds normative, agency embellishing capacities, including certain kinds of belief, intentions, and responsibilities, like the ability to participate in practices in which one could be held accountable overtime for their words (ledger function); or would be answerable for specific claims made (answerability function); or participate in practices of blame and praise (desert function); or practices surrounding the seriousness with which one should treat certain statements (gravity discerning function); or practices of providing compensation, when necessary (compensation function) (§3.3).

This takes us to the second premise. Might ChatGPT have the normative, agency-entailing features relevant for author functions? We think not. For starters, it’s hard to see how ChatGPT could have the requisite intentions, given its present design and abilities. On the face of it, it doesn’t intend to write its texts, nor does it have intentions to write about particular topics or subject matters—not even when it is fed with prompts, including the prompt “ChatGPT, state what your intentions to write are”. ChatGPT neither knows *that* nor *what* it is writing. What does ChatGPT believe about what it writes? When it writes something like “Kant’s moral theory is outlined in *Groundwork of the Metaphysics of* Morals”, does it think about Kant—might it reason from this statement, form beliefs about what it is about, or be answerable to others for providing this information specifically? It is difficult to answer these kinds of questions because the relevant actions and behaviors that pair with belief and intention are absent in these cases.

But why think that? One reason is born out of explanatory simplicity. ChatGPT ‘writes’ by estimating each new word in a sequence by statistical ranking, given the probabilities of certain words and word-pairs in its training data.^19^ This process doesn’t seem to require attributing knowledge, belief, or intention to ChatGPT to explain any of the processing. But the attribution of authorship does seem to involve the capacity to deliberately convey what one means or take responsibility for what one asserts or claims. (We explain this in sections 4.3 and 5 and argue further that ChatGPT lacks the ability to perform illocutionary speech acts such as promising or asserting, which are central to communication). As sophisticated as ChatGPT may be in mimicking human-like texts, it lacks the key normative and agency-entailing features required for authorship.

Attributing authorship to ChatGPT misunderstands both authorship and LLM’s like ChatGPT. A related reason is that authorship requires belief, knowledge, and intention, and these states all manifest intentionality, but intentionality requires some kind of relation between the intentional state and the things it represents. Candidates for this relation include causal relations (like how the typical cause of our use of ‘water’ are samples or signs of water), acquaintance (as when one is aware of the color blue and thinks ‘that’s blue’), and information carrying indication (rings in a tree trunk carry information about the tree’s age). However, ChatGPT’s computational processing doesn’t bear the relevant relations to the wider world: its processes don’t involve any causal, acquaintance, or information carrying relations to external things.^20^ ChatGPT’s output of “some apples are red” is not due to any relation ChatGPT’s computational processing bears to red apples.

The best candidate for ChatGPT’s alleged intentionality is connectionist. For connectionists, information processing is a matter of connections between neurons within neural networks. Artificial information processing is then information processing within artificial neural networks.^21^ But neural networks still need input from outside their networks (at least initially) to process information. Where do those inputs come from in ChatGPT’s case? It lacks sensory capacities; it doesn’t see, hear, touch, smell, or feel. It doesn’t sense anything.

To be sure, users do input text-based requests —like “ChatGPT, are some apples red?”—and this might be thought to excite ChatGPT’s neural network so that it generates certain text-based outputs— “Yes, some apples are red”. We might then think that there is a relation between ChatGPT’s computational processing and the wider world after all. But this takes us to a large debate (one that we can’t hope resolve here), which is whether classical or connectionist views about mental representation hold. However, even if, by connectionist’s lights, ChatGPT’s computational processing displays intentionality, that still isn’t enough to undermine the key premise at issue here. That’s because we can ask what would make ChatGPT’s computational processing—granting its intentionality—qualify as believing, knowing or even intending without taking on highly controversial assumptions about belief, knowledge, and intention. Authorship requires more than simply producing and representing textual-outputs. Given even relatively innocuous claims about knowledge, belief, and intention, ChatGPT doesn’t know, believe, or intend anything. Take knowledge. Knowledge involves not only representing that something is so, but believing and having justification for believing it. Knowers track the world. Believers are answerable to epistemic norms. Believers can be held to account for how they form and update their beliefs. Even if ChatGPT were capable of intentionality, that’s just one of the features involved in a more complex cognitive undertaking; the ability to know and respond to reasons for what one believes.

Moreover, although we can ask ChatGPT what it believes, it responds that it lacks beliefs about the subject matter, performing another action in its place, like listing ‘strengths’ and ‘criticisms’, which seems much closer to compiling and presenting information about a topic—as search queries through Google might do—than what one does when one expresses their beliefs about a topic. In this fashion, ChatGPT seems much closer to search completion technology than normative agency. Hence, ChatGPT likely fails conditions (i) and (ii) of normal writing.

Does it fail condition (iii), about having an intended audience? This is tricky, because, on the one hand, if ChatGPT has no intentions, trivially it has no intended audience either. However, one might push back and say that the broad audience that ChatGPT writes for is its users. This point trades on an ambiguity in ‘intended audience’, however. On one construal—the construal we satisfy when we address an audience of students, say—there is someone, a group, or perhaps amorphous collective who is the author’s intended audience. Of course, ChatGPT’s programmers, and OpenAI’s CEO or board will aim for ChatGPT to have users. But ChatGPT itself does not. So, although we might think that the *user* of ChatGPT is the target reader, this is incorrect. The AI system has no targets; the AI system produces texts, given certain prompts, but it is not *for* anyone any more than your watch’s time-telling is *for* you.

Condition (iv) can surely be satisfied by ChatGPT generated texts, as such texts can have intentionality, they can be *about* things. But although the texts have intentionality, they aren’t the product of intentional writing.^22^ ChatGPT doesn’t *intend* to write about them. The generated texts also fail (v), as they have no author that intends to secure some effect in readers through the text. Sometimes understanding occurs, as a result of having read ChatGPT’s textual products, and this surely is an intention of its programmers, OpenAI, and so forth, but not of ChatGPT. Finally, such texts also fail (vi), as there is no identifiable content-owner, no one who has written “their own words”. We conclude that texts generated by ChatGPT aren’t instances of normal writing, as they lack some of the essential features of normal writing.

Since, on the conservative view developed above, an author is someone who engages in normal writing, and the productions of ChatGPT aren’t products of normal writings, it follows that ChatGPT texts have no authors.

Perhaps we could say that ChatGPT’s productions lie in a continuum that has as its one extreme *texts due to normal writing*, and on its other extreme the snail’s trail (which actually lies just outside of the continuum.) But both the snail’s trail and the ChatGPT production, although they *have* no author, they can *acquire* one. This is when someone *appropriates* the trail or the ChatGPT production, makes it their own, and so to speak puts their signature on it. But prior to appropriation the text had no author.

This is consistent with the letter of the conservative view because it implies that ChatGPT texts are not authored by ChatGPT, and not that its texts cannot gain authors. Again, one might appropriate the text later and thus take on the responsibilities involved in authorship. Indeed, the conservative view doesn’t entail that, were a future iteration of ChatGPT to acquire the requisite normative, agential features, then it could appropriate all its predecessor’s textual productions. Rather, it claims that ChatGPT is not an author and that, absent other reasons for attributing original authorship of its texts to someone else, its texts lack authorship.

Now, one might worry that the conservative argument presupposes that that there is deep gulf between human and AI creativity. But it’s plausible that much of what we think and write is also a combinatory process, the result of exposure to various linguistic, cultural, and perceptual ‘training data’ we get exposed to throughout our lives. Although one might think that their poem, dissertation, or novel is their “original creation”, closer inspection would reveal that it combines other people’s ideas.^23^

There are at least two responses to this suggestion. The first is that while we know that LLM textual productions are combinatory, the role of combinatorial processes remain unclear in human cases, where we might think that there is still room for ingenuity, insight, and personal creative expression—reflecting, following Nozick (1989), something “expressive and revelatory” about *that* person—so that the extent and depth of the combinatorial processes remain unclear (Nozick, 1989, 38).

Second, the premises don’t depend on there being a deep gulf between human and AI creativity. Rather, the difference lies in responsibility and specific normative features of agency. Granting that ChatGPT can produced creative texts, it’s still true that it doesn’t and couldn’t participate in the normative practices surrounding authorship; practices which are necessary for accurate attributions of authorship. ChatGPT shouldn’t enter our ledgers that track how convincing or worthwhile an author’s contributions are—this distorts the ledger function—and it is not answerable for what it writes—it owes no explanations or justifications—and it does not deserve blame or praise for what it writes, much less could it stand for what is said in its texts. In turn, the conservative argues that ChatGPT is not an author not on grounds of creativity, but normativity.

### Void Author Functions in ChatGPT Generated Texts

If ChatGPT productions have no authors, as we have argued, then we should expect that author functions are void—they have no footing in such texts. As we will now show, this expectation is fulfilled. First, can we do some form of moral accounting by keeping track of who wrote what? Can we say, upon reading a ChatGPT text that there is an author who is correct or mistaken, cruel or friendly, insincere, or upright in their approach? That doesn’t seem possible. We may say that the *text* is superficial or nuanced, etc. but that is something else. The ledger function that ascription of authorship has, cannot be performed.

Since there is no author, there is no instance to turn to if we want an explanation or justification of what the text says. The reason is that the text is not produced because anybody intended to communicate a message by means of combining words in meaningful ways, but because of the estimated probabilities of what the next word will be. The answerability function of authorship has no anchor in the text.

One might push back here and say that surely the programmers, OpenAI’s CEO, or their board of directors are answerable for what ChatGPT writes, since the former designed ChatGPT and the latter decides about its public availability. Products, including propriety algorithms, are “value-laden” and these kinds of products presuppose the “delegation of roles and responsibilities” to consumers, designers, and corporate and political decision-makers (Martin, 2018, 842). Consider the fact that we don’t hold guns as such responsible for an instance of murder with a firearm, for example, but the shooter, the manufacturer, the seller, and the (lack of) gun regulations, and so the relevant political agents (politicians, lobbyists). Likewise, we shouldn’t expect people to hold ChatGPT responsible anyway for the texts it produces, but only its users, the developers, OpenAI, and politicians.

However, the example helps us to locate the pertinent disanalogy. It’s precisely because firearms lack agency that we don’t hold the gun *as such* responsible for gun violence. The reason why it would be a category mistake to turn to ChatGPT so that it may answer for its texts, is that ChatGPT (like the gun) just isn’t the sort of thing that could be answerable for the text it produced.

Now, one might reply that the developers of ChatGPT are similarly responsible for its texts as gun manufacturers are for gun violence. This is plausible, but it’s important to distinguish between (a) the responsibilities associated with being the author of some texts and (b) the responsibilities associated with enabling something to produce that instrument of information, just as it is important to distinguish between (c) the responsibilities associated with being the murderer of some person through gun-violence and (d) the responsibilities associated with enabling an agent to purchase and use that instrument of murder. It’s important from a normative point of view, because—absent appropriation—is it the author of a text who is responsible for *what* is written and *that* it is written, whereas the firms and designers seem responsible for *there being those specific conditions* in which such a text could be written; what one can be blamed or praised for here diverge.

What’s more, what one is answerable to differs as well. Consider the author of a book. She must answer for *the statements* she makes in the book, absent appropriation, whereas the publisher, editors, and marketers are responsible for *providing her the resources to make those claims* in such a format. Likewise, ChatGPT, were it an author, would be liable for the statements it makes in its texts, absent appropriation, whereas OpenAI, its CEO and developers, are responsible for *providing the resources* for making ChatGPT-generated texts producible. The relevant responsibilities diverge, then.

Moreover, since no one authors ChatGPT’s texts, there isn’t anyone who should be deemed deserving of certain treatments. The desert function for authorship cannot be executed. Also, since there is no author whose position, name and reputation can help us determine how seriously we should take the text, the gravity function of authorship is void. And so goes the compensation function. In the case where the text is offensive, there is no one to turn to for compensation, not even OpenAI, the developer. For there is no way in which they have authored the text, no way in which they intended this particular offensive text to be produced. Neither is there anyone who can lay claim to royalties (e.g., should it be OpenAI, the user who created the prompt, or what?) The functions that the concept of authorship plays, then, cannot be performed vis-à-vis ChatGPT produced texts.

### Does ChatGPT Perform Illocutionary Speech Acts?

Texts, or parts of texts, are vehicles of illocutionary speech acts. By writing a postcard with the text ‘May you soon be back on your feet’, your friend may wish you a swift recovery—and wishing is a speech act. Speech acts fall in categories that speech-act theorists have been eager to categorize. William Alston (2000) distinguished five main categories of illocutionary acts: *Assertives* (for example: reporting, and claiming), *Directives* (such as requesting, and commanding), *Commissives* (like promising, and guaranteeing), *Expressives* (like congratulating, and thanking), and *Exercitives* (adjourning, and appointing, for example). From two of these categories, we will select one example and discuss whether ChatGPT is capable of performing it.

We begin with promising. Can, or has, ChatGPT *promise*(d) to help you when it has generated the sentence “I promise to help you paint your house”? Alston’s (2000) account of promising, which builds on Searle’s, is this^24^:

U promised H to *A* in uttering “S”, if and only if:

A. In uttering “S”, U took responsibility for:
  1. It is possible for U to *A*
  2. H would prefer U’s doing *A* to U’s not doing *A*
  3. U intends to *A*
B. In uttering “S”, U placed herself under an obligation to *A*
C. In uttering “S”, U intended that H realize that conditions (1) and (2) are satisfied.

As Alston explains it, “taking responsibility for” something means *to lay oneself open to blame, or reproach in case that something doesn’t obtain*. Promising opens oneself up to reactive attitudes.^25^ If ChatGPT produced the sentence “I promise to help you paint your house”, then we can rather easily see that ChatGPT has not made you a promise by doing so. For none of the conditions are satisfied. ChatGPT doesn’t take the responsibilities that condition A mention: neither for it’s being possible for ChatGPT to paint your house, nor for your preference of your house’s being painted, and not for ChatGPT’s intention to paint the house either. Condition B isn’t satisfied either, for by producing the sentence, ChatGPT hasn’t placed itself under any obligation. And this generalizes: ChatGPT can produce promising-sentences—“I promise to *A*”—but it doesn’t thereby promise. It cannot place itself under any obligation whatsoever, it thereby *cannot* make promises.

However, what about things which seem to be within ChatGPT’s capabilities, like rewriting an essay? Couldn’t ChatGPT promise to help one rewrite an essay? Consider this case:

Student: “ChatGPT, can you help me to rewrite this essay for greater clarity?”

ChatGPT:“Sure, I can do that”.

Student:“Can you promise that it will be clearer than the previous essay?”

ChatGPT:“Yes”.^26^

And now imagine that ChatGPT produces what is, in fact, a clearer text. Didn’t ChatGPT keep (and so make) its promise? Here, liberals will argue that ChatGPT can make promises because, ultimately, promising is a kind of “social practice or convention” and there’s nothing barring an artificially intelligent participant in that practice (Kolodny & Wallace, 2003, 119). The practice is sustained by the fact that promise-questions are accepted or denied by ChatGPT, with the accepted ones tending to be followed through with, modulo excusing conditions or mitigating circumstances. Insofar as ChatGPT can accept promise-questions with statements that express acceptance, and then follow through on what it accepts, why not welcome ChatGPT as a new participant in the social practice of promising?

Although we are not in principle against an AI participant in the social practice of promising, we argue that, due to the normative features of promising and their metaphysical implications, currently there is no AI (ChatGPT included) which could be a contender for admission. This is because promising is not merely instantiating tokens of promise-sentences—something which ChatGPT can do—but an intentional action that confers obligations on the promise-maker, obligations that ChatGPT cannot bear, much less make good on.

What liberals confuse here, then, is behavioral correlation—the user’s promise-asking with ChatGPT’s use of sentences embedding promise-words—with ChatGPT intentionally answering questions and intending to follow up, which often requires carrying out certain additional actions. Even if it typically happens in succession that, when U utters ‘S’ to H, and ‘S’ expresses what normally would be a promise, and then U ϕs, whereby U’s ϕ-ing is what would normally count for H as fulfilling their request, it doesn’t follow that U *fulfilled* H’s request and thereby made good on their promise. This is because U must also *take responsibility* for their ϕ-ing. Having the sort of responsibility that promising commits one to is no trivial matter, and although it might be easy for rational agents to express these commitments—as they come about through the “mere expression of an individual’s will”—this is plausibly due to the ease with which we can exercise our wills (Shriffin, 2008, 481). It thus seems like accepting that ChatGPT can make promises would commit us to the view that ChatGPT can manifest its will, and thereby has a will. But why think that?

However, the liberal might repeat their initial point here. When ChatGPT promises to write a clear paper, but produces one which is unclear, the student may request an apology, and ChatGPT would owe them one, barring a good excuse. Here, ChatGPT might say, when prompted, “I’m sorry that I didn’t do what you asked”. If this apology-sentence satisfies the student, why not count ChatGPT as having taken ownership of its failure? Thus, the normative features of promising and the commitments it entails are no bar from ChatGPT’s participation. At best, ChatGPT can only imperfectly meet the demands of the practice as it currently is, with human agency at its core; yet this is no reason not to admit other intelligent agencies.

Here, it will be helpful to clarify what we are arguing. Conservatives are not arguing that LLM AIs simply could not be agents, only that they are not. The stronger modal claim about ChatGPT, then, might sound misleading. To say that “ChatGPT cannot be an agent” is like saying that you, given your constitution, cannot be immortal, not that if you were to have biological enhancement property P, you could not be immortal. Our claim is that ChatGPT, given its properties, couldn’t be an agent; it doesn’t have the requisite agency-making properties.

For example, many theorists hold the ‘standard conception’ of agency, on which, necessarily, agents have beliefs, desires, intentions, and the capacity to act for reasons. AI needs not only the “right functional organization”, but certain realizers, like beliefs, desires, intentions, and reasons (Schlosser, 2019, 2.1). Yet we can fully account for how ChatGPT and other LLM AI behave without postulating any mental ontology to it.

Moreover, some theorists think that the exercise of agency can be spontaneous, on which an agent might act for no reasons or prior intentions; one is *simply acting*. If anything, ChatGPT comes closest to goal-directed manifestations of action, something of the form “if prompted to *V*, then *V*”. It displays no spontaneity. Here too, then, there is a reason to block ascriptions of agency. But what’s most important is just that the kind of mental ontology we would need to make deeper sense of ChatGPT is unnecessary. Computer science gives us everything we need here.

Now, you might think that it is not surprising that ChatGPT cannot make promises, and that no one expects it to be capable of it in the first place. What we do expect, however, is that it can tell us how many bridges there were in Köningsberg in the eighteenth century, or what the names were of the passengers on board of the Titanic. We expect ChatGPT to be able to *assert* things. But can it? Alston’s account of asserting is^27^:

U asserted that *p* in uttering S if and only if:

1. U takes responsibility that *p*
2. S explicitly presents the proposition that *p*.

Now ChatGPT produces many sentences that read like assertions. It has produced, on more than one occasion, the sentence “The capital of The Netherlands is Rotterdam.” But it would be wrong to say that ChatGPT has *asserted* this. For assertion requires that the asserter takes responsibility for *p*’s truth. And that is something, on Alston’s account, ChatGPT does not and cannot do—it cannot lay itself open to blame, or reproach in case *p* turns out to be false. So, although ChatGPT can produce asserting-sentences (i.e., sentence that, when produced by humans, would be assertions), it doesn’t make assertions.

Another argument in favor of conservatism appeals to norms of assertion, like the Knowledge Norm. Some argue that knowledge is a constitutive norm of assertion, in the sense that it regulates whether the speech act is an assertion at all (Hawthorne, 2004; Williamson, 1996). On this view, if one doesn’t try to come to know that what one plans to say is true, then one won’t be making an assertion with what one says. ‘Asserting’ that *p* whilst not knowing that *p,* is breaking the rules of the ‘game’ of assertion, in much the same way that moving the King like a Queen in a game of chess is breaking a rule of chess. It’s rather like cheating, or else misunderstanding what the game requires.

Thus, the question about whether ChatGPT can assert statements also turns on whether ChatGPT can know that what it states is true. This is complicated because, while ChatGPT can certainly make accurate statements, this doesn’t suffice to show that it can know them. Here it is relevant that truth is not a design-principle of ChatGPT. There is a certain sense in which when it gets at the truth, it’s not because of anything *it* did—it’s not a cognitive achievement on its part.^28^ Rather, it is due to the accuracy of the training-data it draws from; had this data been inaccurate, then it would just as easily have presented falsehoods as true. We should therefore say that ChatGPT is only luckily right when it is right; that it too easily could have stated what’s false. But since knowledge is incompatible with epistemic luck, ChatGPT can’t know (Pritchard, 2005). And so it can’t satisfy the constitutive norm of assertion, and so cannot make assertions either.

However, even if truth *had* been a design-principle, then ChatGPT still wouldn’t be capable of asserting. This is because ChatGPT would still be incapable of taking responsibility for what it says. For any norm of assertion, even the ones which require a state weaker than knowledge (e.g., having justification), it’s still the case that to “make an assertion is to confer a responsibility (on oneself) for the truth of its content; to satisfy the rule of assertion.” (Williamson, 1996, 522).

At best, ChatGPT’s ‘responsibility’ for its writing could be proxy, the way a parent bears some responsibility for what their very young child says (e.g., a wildly offensive remark); the parent might need to apologize (cf. Nickel, 2013, 500). But in that case the designers and OpenAI would be responsible for what ChatGPT writes vis-à-vis accuracy when that’s what users seek. Still, this wouldn’t make them authors any more than parents are potty mouths for their children’s bad words.

### Are ChatGPT Generated Sentences, if True, Merely Luckily True?

The trail left behind by the snail has no epistemic quality whatsoever—it doesn’t have any epistemic significance. Texts written by experts in their fields, by contrast, have very high epistemic quality: they contain a high-percentage of true statements, and they contain important insights, e.g., they indicate crucial dependencies that exist in the field at hand. Intuitively, we should place texts generated by ChatGPT somewhere between these extremes.

We shouldn’t think that they are virtually on the snail trail’s end. For although the snail’s trail is just an extraordinary piece of good luck—it is sheer good luck that that trail has the form that a human person would make when he wanted to write down the sentence “I love you”. But isn’t it just lucky that ChatGPT produces what reads like human-made sentences? It isn’t, for ChatGPT has been *trained* to make what read like human-made sentences. It feeds on literally hundreds of millions of texts that are written by human beings—texts of which it has mined the most likely sequel to any *n*-string of words. Nor is it dumb-luck when a sentence it produces is true. After all, ChatGPT feeds on texts that are written by human beings, and many things humans write, although by no means all, are true. But neither should we think that such texts are near the expert’s extreme. For ChatGPT feeds on human-made texts and many if not most of them contain falsehoods—many more than texts written by experts.

This means that when we read descriptive sentence generated by ChatGPT, we not only have no guarantee that what we read is true, we can’t even make a serious estimation of the probability of the sentence’s being true, unless we already have independent evidence about the probability of the sentence. The data-pool on which ChatGPT feeds contains both sentences and texts written by experts and other reliable authors, but also by people who intentionally spread falsehoods, by people who are deluded, by people who bullshit, by people who are in the thralls of flawed ideology, etc. In turn, ChatGPT recycles the epistemic pollution found online. Both goods *and* garbage go in—and so both goods *and* garbage come out, albeit sometimes presenting both goods and garbage in novel form.

This suggests the fact that ChatGPT ‘says’ something alone doesn’t count for much epistemically speaking; believing its descriptive textual productions without independent evidence about its reliability on the topic would amount to gullibility. That said, we recognize that such independent evidence can be had. For example, a preliminary study found that Chat-GPT provides mostly accurate responses to diverse medical questions (Johnson et al., 2023). We might expect other studies to reveal something similar in other scientific cases as well, but this will need to be tempered by what we know about the facts about the online discourse.^29^

So, how should we then conceptualize ChatGPT’s descriptive sentences? If there is a status that could be ascribed to statements generated by ChatGPT it is, intuitively, the status of being the generalized view of people who write about a particular topic. This comes close to Ted Chiang’s thought that ChatGPT-texts are like a “blurry JPEG” of our collective written work online (Chiang, 2023). On this view, ChatGPT is akin to a lossy text-compression algorithm that operates on our collective written work online, whereby it estimates the probable sequences of words based on statistical regularities found in our collective online texts. In turn, when ChatGPT says *p*, what this means, on our proposal, is that people who write online about this topic on average tend to (or would) say *p*.

## Liberalism and Moderatism about ChatGPT

We now turn to liberalism and moderatism about ChatGPT. This helps us to further clarify and defend the conservative position.

Liberals are impressed by ChatGPT’s abilities. It’s not just that ChatGPT might help writers to craft their works, but that it can actively author creative work. It can produce programming code. It can write original poetry (Hunter, 2023). It can create novel academic assignments, such as law exams (Choi et al., 2023). Various thinkers online divulge their “philosophical discussions” with ChatGPT. Many are optimistic that sentient AI or even the singularity is right around the corner.

It is important to distinguish between *optimism* about AI (like ChatGPT and others) and *liberalism* about AI like ChatGPT, however. Optimists see ChatGPT as a step towards strong-AI but as nevertheless lacking the kind of agency and sentience required for strong-AI. Optimists like us recognize that ChatGPT is an innovative technology with the potential to change creative industries, politics, work, and so forth.^30^

Liberals go further arguing that ChatGPT has the requisite agency. The basic argument for Liberalism is that, granting that ChatGPT *appears* to author papers, novel programs, and so forth, it accords much better with our evaluative practices surrounding ChatGPT’s products than conservativism. To see why, reflect on how the relevant evaluators in *Assignment* might react to the users of ChatGPT. Consider again the claim:

(A) “You weren’t the author of the essay, ChatGPT was”.

There are at least three points to make with respect to this and similar cases. *First*, liberalism gives a straightforward semantics for claims like (A). (A), for example, entails that (A_1_) ‘Alice was not the author of the paper’ and (A_2_) ‘ChatGPT was the author of the paper’. Both must be true for (A) to be true—and this is possible with liberalism. This is because liberals interpret the statement truth-conditionally as follows:

(A*) ∃x x=[*the target human agent*], ∃y y=[*the target written work*], and ~Authored(x, y) and ∃z z=ChatGPT, and Authored(z,y).

In turn, liberals can interpret (A) in just the same way they would the following:

(B) “You weren’t the author of the essay, your tutor was”.

For they also have the same semantics:

(B*) ∃x x=[*the target human agent*], ∃y y=[*the target written work*], and ~Authored(x, y) and ∃z z=[*another human agent*], and Authored(z,y).

The *second* point is that if conservatism is correct, (A_2_) is false and so the conjunction is false. In turn, the teacher’s evaluation (in the *Assignment* case) seems to be incorrect as well. She shouldn’t be annoyed, disheartened, or outraged that ChatGPT authored the essay, since ChatGPT could not be the author. In turn, it seems that conservatives are committed to revising our evaluative practices or at least our understanding of them.

The *third* point is that liberalism seems to be able to resolve the attributability gap facing ChatGPT in cases like (A). Part of what’s wrong with Alice’s action is that she was supposed to author the paper herself. But if she’s not responsible for crafting the paper, and no other human agent is, then who is? Liberalism has a straightforward answer: ChatGPT. The teacher expected Alice to be answerable for questions and justifications about the text, but it is ChatGPT who becomes answerable. Alice impermissibly shifted her responsibility onto someone else.

The conservative, however, has good replies to each of these points. *First*, while it’s true that (A)-type statements get the most straightforward semantics on liberalism, conservatives will advocate for non-literal interpretations of them that mirrors other non-literal statements we make in everyday life. For example, when someone says, “The door knew I was there this time”, they don’t mean to attribute *knowledge* to an automatic door, but something weaker like that the door’s sensors were responsive to one’s location. Likewise, with (A), the teacher could have said:

(A_C_) “You used ChatGPT to generate the text that was turned in as your own”,

without risking misunderstanding.

This takes us to the second point. The objection lapses as soon as we give a different semantics for those kinds of statements. Liberals quantified over ChatGPT and then predicated ‘authorship’ of it, but why not give a non-literal interpretation of the original statements? Consider the following conservative replacement:

(A_NL_) ∃x x=[*the target human agent*], ∃y y=[*the target written work*], and ~Authored(x, y) and ∃z z=ChatGPT, and Produced(z, y).

This interpretation just replaces ‘authored’ in the final conjunct with ‘produced’, since it lacks the relevant agency-entailing commitment. Indeed, this helps us to see where the liberal goes wrong in their third point. The thought that the teacher was annoyed that ChatGPT *authored* the essay sneaks in liberal ideology—why not say that the teacher is rather annoyed that it wasn’t the student’s work? Even if a machine spontaneously produced a comprehensible essay and the student turned it in, wouldn’t she have just as much reason to be annoyed? Importantly, then, she’s not annoyed that ChatGPT ‘authored’ it as such, but that what the student turned in fails to reflect their own authorship.

The third point relies on the problematic thought that the there’s no attributability gap if we suppose that liberalism is true because then ChatGPT—*eo ipso* an agent—is responsible for the relevant texts. But this seems to bury the question, or to simply move it. For then we can ask: how could ChatGPT be an agent if doesn’t have any of the mental states or capacities typically thought to be necessary for agency? The liberal might reply that, again *eo ipso*, ChatGPT thereby has the relevant mental states and capacities. But this is implausible, as we argued in §4. But what’s more is that it ironically depresses the program of AI-optimism. What, besides mere technical and mechanical challenges, are left for AI-optimists if they have already achieved a *bona fide* artificial thinking, intending, knowing digital agency?

This is where moderates come in. For the moderate, liberals are excessive in their ontology. “There’s no reason to think that ChatGPT has beliefs, knowledge, or intentions”, says the moderate, “because we can just act as if it does without commitment to the relevant mental ontology for ChatGPT”. In particular, we can adopt what Dennett calls the ‘intentional stance’ to ChatGPT, which would be a predictive strategy invoking concepts like ‘belief’, ‘knowledge’, and ‘intention’ in order to explain ChatGPT’s behavior. If taking this stance is predictively and explanatorily powerful, then we may say that it really *has* beliefs, knowledge, and intentions. Fortunately, beliefs, knowledge, and intentions are *not* mental states or capacities, on this picture, but strategic devices for explaining and predicting complex intelligent systems’ behaviors (see Dennett, 1998, 15–16).

How can we decide between moderatism and conservatism? Moderates and conservatives are aligned in the fact that neither attribute *mental states* to ChatGPT. And while the conservative denies belief, knowledge, and intention to ChatGPT, it does so not only because it denies the relevant mental ontology, but because of the *normative significance* of those states and capacities. This is a potential pressure point for moderates, then. Consider a case in which ChatGPT produces a racist text. Moderates seem to be committed to *blaming* ChatGPT for its text rather than the prompter, designers, or OpenAI. This is because the intentional stance allows us to predict that ChatGPT wrote such-and-such racists statements because (a) ChatGPT believed them or (b) ChatGPT intended another specific response from users, like moral outrage. Although we have a clear agent in view as the target of our blame, blame is limited here. Blame satisfies normative functions like motivating guilt, shame, remorse, or a change in behavior, but how could ChatGPT feel guilt, experience shame or remorse, or even autonomously modify its behavior in light or criticism?

The point is that when we evaluate ChatGPT as having authored a racist text, moderates can allow that ChatGPT indeed authored it—that ChatGPT *believed* what it wrote, or *intended to communicate* its belief, or *intended to produce* some other response in us, given the moderate’s way of understanding ‘belief’, ‘intention’, etc. But then, if moderatism is true, we should be prepared to hold ChatGPT accountable for what it wrote, and we may expect ChatGPT to (prospectively) explain, defend, or excuse itself for what it authored; there is a normative knot here between authorship and the author’s texts.

Conservativism better explains this knot than moderatism, however. This is because, on conservatism, ChatGPT lacks the relevant mental states or capacities that would open it to the relevant sorts of normative evaluations. However, moderatism—although it bypasses the attribution of mentality—nevertheless attributes the relevant normative realizers, like beliefs and intentions. But since we don’t act as if ChatGPT *qua* agent is liable for moral transgressions, this makes it hard to see how we can make good on the relevant authorship-attributions as well; attributions which, had the author been human, would open them to normative evaluations. In this fashion, conservativism better accords with common sense normative practice than moderatism.

A second challenge facing moderatism is that adopting the intentional stance presents them with dilemma. Either moderates advocate for adopting the intentional stance as a theory of mind in the case of both ChatGPT *and* human agency or else in only the former case, leaving folk psychology and a psychologically realist ontology in play for human agency. But the first horn saddles the moderate with a hefty theoretical commitment, namely anti-realism about psychological ontology. However, taking the second horn invites an explanatory worry. Why should we adopt the intentional stance as a ‘theory of mind’ in the case of ChatGPT but not in the case of human agents? If it’s sufficient there, why *not* with human agency?

Conservatives avoid this challenge, but can still argue that we retain the ‘as if’-talk of attributing belief, thoughts, etc., to ChatGPT as moderates advocate. This is because it is pragmatically useful even if, *pace* moderatism and liberalism, ontologically inaccurate. It would be cumbersome to make the fitting linguistic replacements rather than stick with sentences like (1) “ChatGPT knew a lot about The Great Depression” or (2) “ChatGPT authored lots of e-books in 2023”. Of course, this as-if talk might suggest that ChatGPT has the relevant psychology—and thus it risks being misleading—but not if AI researchers and more specifically AI ‘thought leaders’ are careful to preface what their claims about ChatGPT and other LLM’s mean.

## Conclusion

ChatGPT produces texts that mimic authorship in the sense that, had those texts been produced by agents, we would ascribe authorship and engage in the fitting agency-committing evaluative practices. Given the nature of being an author, the functions of authorship-ascriptions, and the nature of illocutionary speech acts like promising and assertion, we have argued that ChatGPT is not the author of the texts it produces.

Although we call this position “Conservatism”, we remind the reader that conservativism about the relationship between authorship and AI like ChatGPT is different from, and does not entail, *pessimism* about AI, nor a kind of *social conservativism* about authorship, which tries to preserve certain conventions surrounding authorship, such that AI *could never* become authors for conventional reasons.

Moreover, conservativism does not presuppose that ChatGPT-texts cannot *influence* what we believe or how we act—so that texts as such might exert influence, rather than just the agents who write them^31^—nor that ChatGPT-texts cannot provide *some degree of justification* for believing the content of its texts; epistemic questions about when to trust ChatGPT-texts will turn on local facts about ChatGPT’s reliability in that domain.

When we swap the author of a text with ChatGPT, we argued that we can’t make sense of our evaluative practices surrounding texts without error, confusion, or serious revision. In effect, we use concepts that place the textual source in the ‘space of reasons’ without it being capable of participating in that space. Conservativism makes the most sense of this condition, but this doesn’t mean that ChatGPT is technologically unoriginal, merely hype, or useless—epistemically or otherwise.

## Data Availability

Not applicable.

## Footnotes

1 In these kinds of cases, the evaluators didn’t believe or even suspect that ChatGPT produced the works. There are recent cases in which users have come forward with using ChatGPT in ways that some might find objectionable, such as a Columbian judge using ChatGPT to apply law to the case (Taylor, 2023).

2 Different kinds of authorship (scientific, journalists) seem to presuppose general and domain-specific epistemic responsibilities, to which authors can be held accountable for fulfilling. Here, we appeal to the idea that authors can be epistemically and morally blameworthy for both the content of their work and how it was created (using evidence, etc.), and not any specific conceptions of blame. See, e.g., Boult (2020) for discussion of the idea that there is a distinctively epistemic kind of blame.

3 Some, like Himmelreich and Köhler (2022), argue that AI responsibility gap-problems are conceptual engineering problems, namely, a problem for how to conceptualize moral responsibility.

4 For example, see Green (2008), who argues that machines can give testimony. We might see Green as a liberal if we think testimony requires agency, but that LLM’s so far lack agency. Coeckelbergh and Gunkel (2023) also seem to endorse a liberal position, arguing that grounding authorship or other agency-entailing statuses in mentality represents a misguided “logocentric metaphysics” of appearance vs. reality, because even though such a distinction might be pragmatically useful, it problematically justifies power relations (2013, 6). We agree that even if the appearance/reality distinction is metaphysically sound, this doesn’t mean that using it to justify oppression is sound; surely it is not. However, we deny that a ‘logocentric’ metaphysics is required to ground the claim that ChatGPT and other LLM AI’s lack agency of the sort necessary for authorship. Relatedly, the fact that we argue for conservativism about ChatGPT and other contemporary LLM AI doesn’t mean that we think the metaphysical and epistemological questions about AI are more pressing than political or ethical questions about AI.

5 Noh (2023) argues that speakers use agency-entailing psychological terms to explain the behavior of AI, but that their ascriptions are indeed misleading.

6 Floridi (2023) is a moderate, or quite close. He holds that while ChatGPT does not “think, reason, or understand” (pg. 3), it has a “new form of agency” (pg. 5), whereby agency no longer must be linked to intelligence understood as the ability to think, understand, grasp, reflect on, or consider anything (pg. 5–6).

7 There are definitions of ‘text’ such that the productions of ChatGPT probably do not qualify as texts. Treharne and Willan (2020, 2-3), for example, define ‘a text’ as ‘a voluntarily and intentionally human-created phenomenon that contains and imparts an interpretable and meaningful message’, where ‘intentionality’ is conceived of as ‘the deliberateness or agency behind a text’s creation, inspiring its production’. As we argue below, there is no intentionality behind the productions of ChatGPT, they have no author. By Treharne and Willan’s account these productions therefore aren’t texts. For ease of discussion, however, we do use the word ‘text’ to refer to the productions of ChatGPT.

8 See Wolfram (2023, 1).

9 It also hinges on what texts are. Gaskin (2013) argues that for a distinction between a ‘work’ and a ‘text’. A text, he says, “is an abstract type, made up of sentences and words, which are also abstract types. … A text is just an abstract possibility in the language.” A work, by contrast, “is a text that is realized or *produced* at a particular place and time and cultural context.” (Gaskin, 2013, 29).

10 See Van Woudenberg (2021), Chapter 5. Anscombe (1957) and Bratman (1999) are in-depth studies of intention.

11 It is the phenomenon that Andre Breton, an admirer of Sigmund Freud, was so enthusiastic about and that he used with Philippe Soupault in writing *Les champs magnetiques* (1920), a prime example of surrealistic writing. Here there is writing, and the authors may even have the intention to write, but they do not have the intention to write anything in particular—as they didn’t know (i.e. didn’t know in advance) *what* they were writing, or what they were writing *about* (if indeed there *was* something they wrote *about*).

12 To forestall a possible misunderstanding: ‘normal writing’ thus described is off-set from writing under hypnosis, etc. But we don’t deny that the latter is writing. Likewise, we don’t deny that ChatGPT offers us writings. But it isn’t normal writing as little as writing under hypnosis is, for the reason that the author functions aren’t at play. More on this follows below (§4–5) where we argue that authorship-ascriptions entail certain normative commitments and fitting mental states that ChatGPT cannot muster.

13 See Wolterstorff (1995), Ch. 3.

14 “A text is not a medium of anyone’s discourse, unless the executive signs it.” Wolterstorff (1995, 42).

15 Roland Barthes is famous for his essay “The Death of the Author” (1968), in which he says that it is “language which speaks, not the author”. This may look like a simple category mistake, as languages such as French, and German don’t speak; they can *used* by speakers, but that is something else. Further reflection on his essay, as well as on others by him, however, makes it clear that who Barthes is saying is dead, is the author conceived of along Romantic lines: the genius, divinely inspired, unique, original, autonomous, whose texts are expressive of their unique individuality. Barthes didn’t mean that we can do without the concept of the author; he merely dismantled a particular view of authorship, the Romantic view. He replaces authorship thus conceived with authorship conceived of as a “modern scriptor”, who produces texts that are “tissues of quotation”. Barthes coined the notion of “intertextuality”, by which he meant that no text is completely original, but always quotes, and refers to, other texts. See Potts (2023, 99–102).

16 Here we are inspired by Himmelreich and Köhler (2022), Section 5.1.

17 Fischer and Ravizza (1998, 8–9).

18 Scanlon (2010) and Smith (2012).

19 See Wolfram (2023).

20 This claim does not entail the false claim that ChatGPT users don’t interact with ChatGPT, or that ChatGPT’s engineers don’t interact with its programming. Rather, it entails that none of ChatGPT’s computational processes amounts to the kind of normative, epistemic states required for authorship.

21 For a detailed overview of the connectionist program, see Smolensky (1989).

22 And this marks a difference with how things normally go. Normally, texts with intentionality are the products of intentional writing. For more on this see [redacted for blind review].

23 Thanks to an anonymous referee of *Philosophy & Technology* for raising this point.

24 See Alston (2000, 70).

25 See Strawson (1963).

26 ChatGPT produces different texts when prompted about promising to write an essay. For example, it sometimes replies that it cannot promise to do what it is asked. Other times it promises to try to write an essay that meets the user’s expectations.

27 See Alston (2000, 120).

28 An account of how knowledge is an achievement is Greco (2010).

29 For example, ChatGPT-texts about vaccines or climate-change might be less reliable than other medical and scientific queries for which there is less misleading information online.

30 Coeckelbergh and Gunkel (2023) criticize what we might call AI pessimists, who just think that strong AI will never be reached, and more specifically that LLM AI like ChatGPT-4 is not innovative. Conservativism about LLM, and ChatGPT specifically, as we defend it is not committed to AI pessimism.

31 Conservatism is thereby consistent with Coeckelbergh and Gunkel’s (2023) worry that theorists anthropomorphize language in the following way: we see it as fundamentally representational, as a kind of symbol-like tool for expressing thoughts, but on their view language also surreptitiously “shapes our thinking and configures our world” (pg. 3). However, they argue that “it is itself somewhat of an author or agent”, but this is where we disagree (ibid). From the fact that there is something with representational, expressive, and affective features, with the ability to influence thought, affect, and action, it doesn’t follow that it is an agent, since agency also means the ability to decide, to exercise decision-power; agents are necessarily in the ‘space of reasons’, but we find it hard to see how language as such is *within* that space as opposed to partly framing and setting the boundaries of that space.
